# Indocyanine green alters intraoperative parathyroid hormone measurements: a timing-dependent effect

**DOI:** 10.3389/fendo.2026.1981095

**Published:** 2026-10-21

**Authors:** Chia-Hsing Sung, Shih-Ming Huang, Ruei-Jhong Ye, Pei-Jhu Shih

**Affiliations:** 1 Department of Laboratory Medicine, Chang Bing Show Chwan Memorial Hospital, Lugang, Taiwan; 2 Division of Thyroid Surgery, Chang Bing Show Chwan Memorial Hospital, Lugang, Taiwan

**Keywords:** artifacts, immunoassay, indocyanine green, parathyroid hormone, thyroidectomy

## Abstract

**Background:**

Intraoperative indocyanine green (ICG) angiography and intraoperative parathyroid hormone (ioPTH) monitoring are widely used in thyroid surgery, yet analytical interference by ICG remains poorly characterized. This study quantitatively evaluated ICG-induced immunoassay distortions.

**Methods:**

The study included paired *in vitro* spiking experiments (n = 6) and an *in vivo* thyroidectomy cohort (n = 6) with serial sampling during paused surgical dissection. Exploratory cross-platform analysis included paired perioperative samples from one patient and additional *in vitro* testing of three independent specimens.

**Results:**

ICG caused significant false-positive elevations. *In vitro*, the median of individual fold-increases was 5.98 for PTH (1–84) and 3.34 for i-PTH (p = 0.031), producing an abnormal diagnostic ratio inversion (PTH (1–84)/i-PTH > 1.0). *In vivo*, measured levels peaked at 3 minutes post-injection (234.8% of baseline for 1–84 PTH; 146.1% for i-PTH) and approached baseline within 20 to 30 minutes (p > 0.05).

**Conclusion:**

Intravenous ICG can transiently distort ioPTH measurements and may lead to misleading interpretation during thyroid surgery. Whenever feasible, baseline ioPTH should be obtained before ICG administration. The observed return toward baseline within approximately 20 to 30 minutes is preliminary and requires validation before a post-ICG sampling interval can be recommended.

## Introduction

Postoperative hypocalcemia, secondary to inadvertent parathyroid injury, devascularization, or unintended excision, remains one of the most frequent and challenging complications following bilateral thyroid surgery (1). Systematic reviews have firmly established that an early postoperative decline in parathyroid hormone (PTH) serves as a sensitive and reliable predictor of biochemical hypocalcemia (2). Early prediction and standardized biochemical monitoring are essential to distinguish transient from permanent parathyroid failure, a clinical strategy strongly supported by the 2018 American Thyroid Association (ATA) guidelines and established clinical practice frameworks (1, 3).

Given the short circulating half-life of PTH—typically ranging from 3 to 5 minutes—intraoperative or early postoperative PTH testing has become a key tool for surgical decision-making. In endocrine surgery, dynamic biochemical feedback guides critical intraoperative choices, including the need for parathyroid autotransplantation and the timely initiation of prophylactic oral calcium and active vitamin D supplementation. While surgeons routinely rely on kinetic criteria such as the Miami criterion (a >50% decline from baseline within 10 to 20 minutes) to confirm adequate resection in hyperparathyroidism, specific early absolute thresholds or relative percentage declines are widely utilized to predict hypocalcemia risk after thyroidectomy (1, 4, 5).

In parallel, intraoperative management has evolved with the increasing adoption of near-infrared fluorescence imaging. Indocyanine green (ICG) angiography has gained wide acceptance as a real-time navigational tool. Clinical trials and prospective studies show that ICG angiography allows direct anatomical localization and dynamic perfusion assessment of the parathyroid glands, helping surgeons preserve vascular pedicles and reduce the incidence of postoperative hypocalcemia (6–9). However, this combined surgical workflow introduces an unaddressed analytical concern: intraoperative blood sampling for PTH testing frequently coincides with, or occurs shortly after, the intravenous administration of an ICG bolus.

ICG is a water-soluble, amphiphilic tricarbocyanine dye with a molecular weight of 775 Daltons. Following intravenous injection, it binds rapidly and extensively (>98%) to plasma proteins, predominantly lipoproteins and albumin. Due to this high-affinity protein binding, ICG remains largely confined to the intravascular space, emitting fluorescence at approximately 830 nm under near-infrared light. The dye is not metabolized peripherally and is cleared exclusively by the liver into the biliary system (10).

Despite its widespread clinical safety, the potential for circulating ICG to cause analytical interference in optical or luminescence-based immunoassays has not been systematically investigated in clinical settings. Exogenous dye molecules are recognized potential sources of immunoassay distortion through spectral overlap, quenching, or non-specific chemical interactions (11–14). Because ICG forms large complexes with serum albumin and induces distinct spectral shifts (15), it may unpredictably perturb signal generation in chemiluminescence or electrochemiluminescence detection systems (16). In the operating room, undetected positive bias in measured PTH could lead surgeons to overestimate parathyroid vascular viability, potentially delaying necessary calcium supplementation or misguiding postoperative discharge planning.

The structural complexity of circulating PTH further complicates analytical interpretation. Endogenous PTH exists as the biologically intact 84-amino acid peptide (PTH 1–84) alongside various inactive N-terminally truncated fragments (such as PTH 7–84) (17). Second-generation intact PTH (i-PTH) assays capture antibodies directed against the mid-region and C-terminus, thereby detecting both full-length PTH (1–84) and truncated fragments. In contrast, third-generation whole PTH assays specifically recognize the extreme N-terminus (amino acids 1–4) to measure only biologically active PTH (1–84) (18, 19). Differential assay susceptibility to exogenous dye-protein aggregates could unpredictably distort absolute values as well as the diagnostic PTH (1–84)/i-PTH ratio on automated electrochemiluminescence immunoassay (ECLIA) platforms.

Therefore, the primary objective of this study was to quantitatively evaluate the analytical interference of intravenous ICG on automated PTH immunoassays using controlled *in vitro* models and *in vivo* intraoperative tracking across distinct surgical settings, following the Clinical and Laboratory Standards Institute (CLSI EP07) guidelines for interference testing (20). Through this approach, we aimed to characterize the temporal profile of ICG-associated PTH interference and to generate preliminary data regarding the interval over which the analytical bias diminishes after ICG administration.

## Materials and methods

### Study design and ethical approval

This prospective experimental and observational study was conducted at a regional teaching hospital in Taiwan. The protocol was approved by the Institutional Review Board (IRB No. 1150313), and all procedures adhered to the Declaration of Helsinki. Written informed consent was obtained from all patients prior to enrollment.

The study consisted of two parts: an *in vitro* spiking experiment to examine direct assay interference, and an *in vivo* study to track intraoperative PTH changes. The *in vivo* phase included a primary cohort undergoing thyroidectomy (n = 6).

### Pre-analytical validation

To verify sample stability and exclude pre-analytical processing artifacts, testing conditions were systematically evaluated. Centrifugation speeds (laboratory protocol vs. manufacturer recommendations) were evaluated across 10 independent patient samples. Sample storage stability (immediate centrifugation vs. 30 minutes and 3 hours at room temperature or on ice) was evaluated on serial aliquots of a representative baseline patient sample. An allowable bias threshold of ≤ ± 25% was predefined to differentiate analytical interference from acceptable pre-analytical variance.

#### Experiment 1: *In Vitro* ICG exposure

*In vitro* spiking models (n = 6): Basal whole blood samples from six patients were split into equal aliquots. One aliquot served as the baseline control, while the paired aliquot was spiked with 0.5 mL of standardized ICG solution (2.5 mg/mL) to evaluate assay response under saturation conditions.

#### Experiment 2: intraoperative sampling

Primary Thyroidectomy Cohort (n = 6): Six patients undergoing elective total or near-total thyroidectomy were enrolled. Hepatic function was evaluated before surgery in all participants; all six patients had liver function test results within the normal range, and none had a clinical history or diagnosis of liver cirrhosis. Baseline intraoperative PTH (ioPTH) samples were obtained from an arterial catheter immediately before intravenous ICG administration (Time 0). ICG was administered through a peripheral venous catheter in the contralateral arm. Serial arterial blood samples were subsequently collected at 3, 6, 9, 15, 20, 25, and 30 minutes after ICG administration. The use of separate vascular access sites in contralateral arms minimized the possibility of direct sample contamination or transient local venous retention of ICG at the injection site. To minimize biological fluctuations related to surgical trauma, all surgical dissection and tissue manipulation of the neck were paused throughout the 30-minute sampling period.

### Immunoassay platforms and exploratory cross-platform comparison

Routine study samples were analyzed on an automated electrochemiluminescence immunoassay (ECLIA) platform (Cobas e601, Roche Diagnostics, Mannheim, Germany) for second-generation intact PTH (i-PTH; reference range 15–65 pg/mL) and third-generation whole PTH (1–84; reference range 14.9–56.9 pg/mL).To explore whether a similar post-ICG effect could be observed with a different analytical system, an exploratory cross-platform comparison was performed using paired baseline (pre-ICG) and post-ICG serum aliquots collected from one patient undergoing routine surgery with intraoperative ICG administration. Aliquots from the same sampling time points were analyzed simultaneously on the Roche Cobas e601 ECLIA platform (ruthenium-based detection) and an automated chemiluminescent microparticle immunoassay (CMIA) platform (Alinity i, Abbott Laboratories, Abbott Park, IL, USA; acridinium-based detection).

### Additional exploratory *in vitro* cross-platform experiment

To further characterize cross-platform analytical responses, three independent clinical whole-blood specimens were evaluated under an additional exploratory *in vitro* condition. For each specimen, 4 mL of whole blood was paired with an aliquot exposed to 0.2 mL of ICG solution (2.5 mg/mL), mixed for 5 minutes, and centrifuged before analysis. Paired specimens without ICG exposure served as baseline comparators. Measurements were performed on the Roche Cobas e601, Abbott Alinity i, and Siemens Atellica IM immunoassay systems.

### Statistical analysis

Statistical analysis was conducted using Python (SciPy and Pandas). Continuous variables were presented as medians. Fold-increases for paired *in vitro* experiments were calculated for each individual sample and reported as the median of individual fold-changes. Paired comparisons between baseline and post-injection time points were evaluated using the Wilcoxon signed-rank test. A PTH (1–84)/i-PTH ratio > 1.0 was defined as a diagnostic ratio inversion. Statistical significance was set at p < 0.05.

## Results

Pre-analytical testing demonstrated robust analyte stability across the evaluated processing conditions (**Table 1**). Differences between standard and internal centrifugation protocols across 10 samples yielded minimal analytical bias, ranging from -2.79% to +4.69% with a median shift of +0.97% (**Table 1**). Similarly, stability assessments across temperature variations (room temperature vs. ice bath) and storage durations (30 minutes and 3 hours) showed no clinically significant sample degradation, with all deviations remaining well within the predefined allowable threshold of ≤ ± 25%. (**Table 1**). These baseline validations confirm that routine sample handling protocols did not introduce pre-analytical artifacts into subsequent PTH determinations.

**Table 1:** Pre-analytical stability validation for PTH measurements.

Validation parameter	Test condition	N	Bias (%)	Clinical acceptance
Centrifugation Speed	Standard vs. Current protocol	10	-2.79 to +4.69 (Range)	Pass
Storage & Temperature	Room temperature, 30 min	1*	-0.83	Pass
Storage & Temperature	Ice bath, 30 min	1*	6.6	Pass
Storage & Temperature	Room temperature, 3 h	1*	-1.08	Pass
Storage & Temperature	Ice bath, 3 h	1*	3.15	Pass

*Note:* Bias was calculated relative to the immediate centrifugation baseline reference value (44.39 pg/mL). The predefined clinical acceptance criterion was an analytical deviation of ≤ ± 25%.

*Storage and temperature stability assessments were performed using serial aliquots prepared from a single representative baseline patient sample.

In the *in vitro* spiking models (n = 6), the direct addition of ICG produced statistically significant false-positive elevations across both assay generations (**Figure 1**). For the third-generation whole PTH (1–84) assay, the median concentration rose from a baseline of 40.19 pg/mL to 235.10 pg/mL, representing a median of individual fold-increases of 5.98-fold (p = 0.031, **Figure 1A**). For the second-generation intact PTH (i-PTH) assay, the median level increased from 36.26 pg/mL to 120.45 pg/mL, corresponding to a median individual increase of 3.34-fold (p = 0.031, **Figure 1B**). Notably, the physiological PTH (1–84)/i-PTH ratio (normally ≤ 1.0) inverted to > 1.0 across all six spiked specimens, demonstrating a disproportionate vulnerability of the third-generation assay to dye-induced interference.

**Figure 1:**
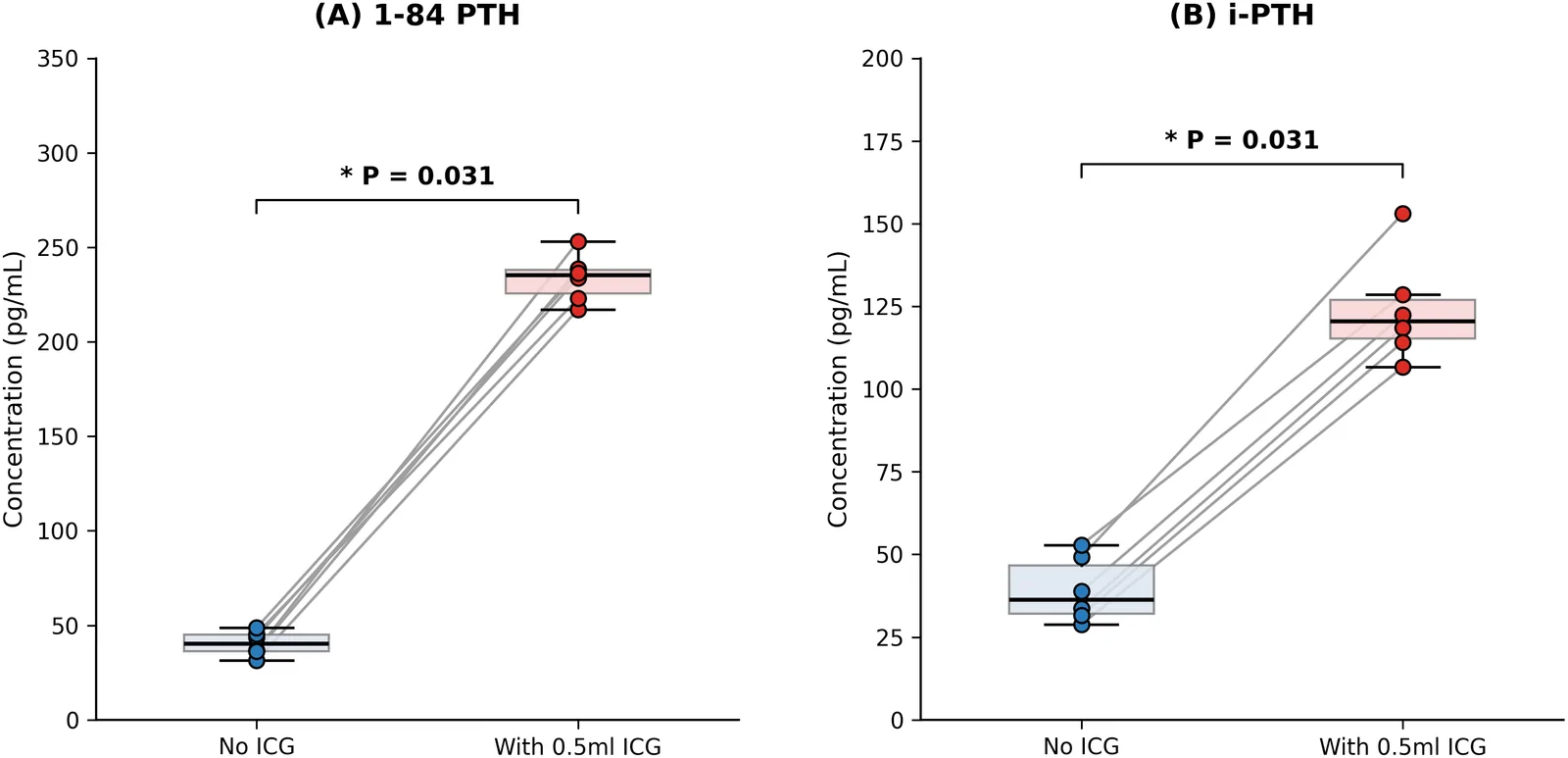
Effect of 0.5 mL ICG addition on measured 1–84 PTH and i-PTH concentrations (n = 6). This image presents a paired comparison of parathyroid hormone absolute concentrations measured without ICG (No ICG) versus with the addition of 0.5 mL ICG (With 0.5 mL ICG) under *in vitro* saturation conditions. Panel **(A)** displays the results for the third-generation 1–84 PTH assay, and Panel **(B)** displays the results for the second-generation i-PTH assay. Spiking resulted in statistically significant artifactual elevations across both assays (*p = 0.031).

During *in vivo* monitoring in the thyroidectomy cohort (n = 6), intravenous ICG injection triggered an immediate and marked, but transient, surge in measured PTH levels (**Figures 2**, **3**). Circulating values peaked at 3 minutes post-injection, with the third-generation PTH (1–84) assay exhibiting a median increase of +50.25 pg/mL (234.8% of baseline; p = 0.031) and the second-generation i-PTH assay showing a median increase of +13.53 pg/mL (146.1% of baseline; p = 0.031) (**Figures 2**, **3**). Although analytical bias attenuated at 6 and 9 minutes, substantial positive interference persisted on both platforms (**Figures 2**, **3**).

**Figure 2:**
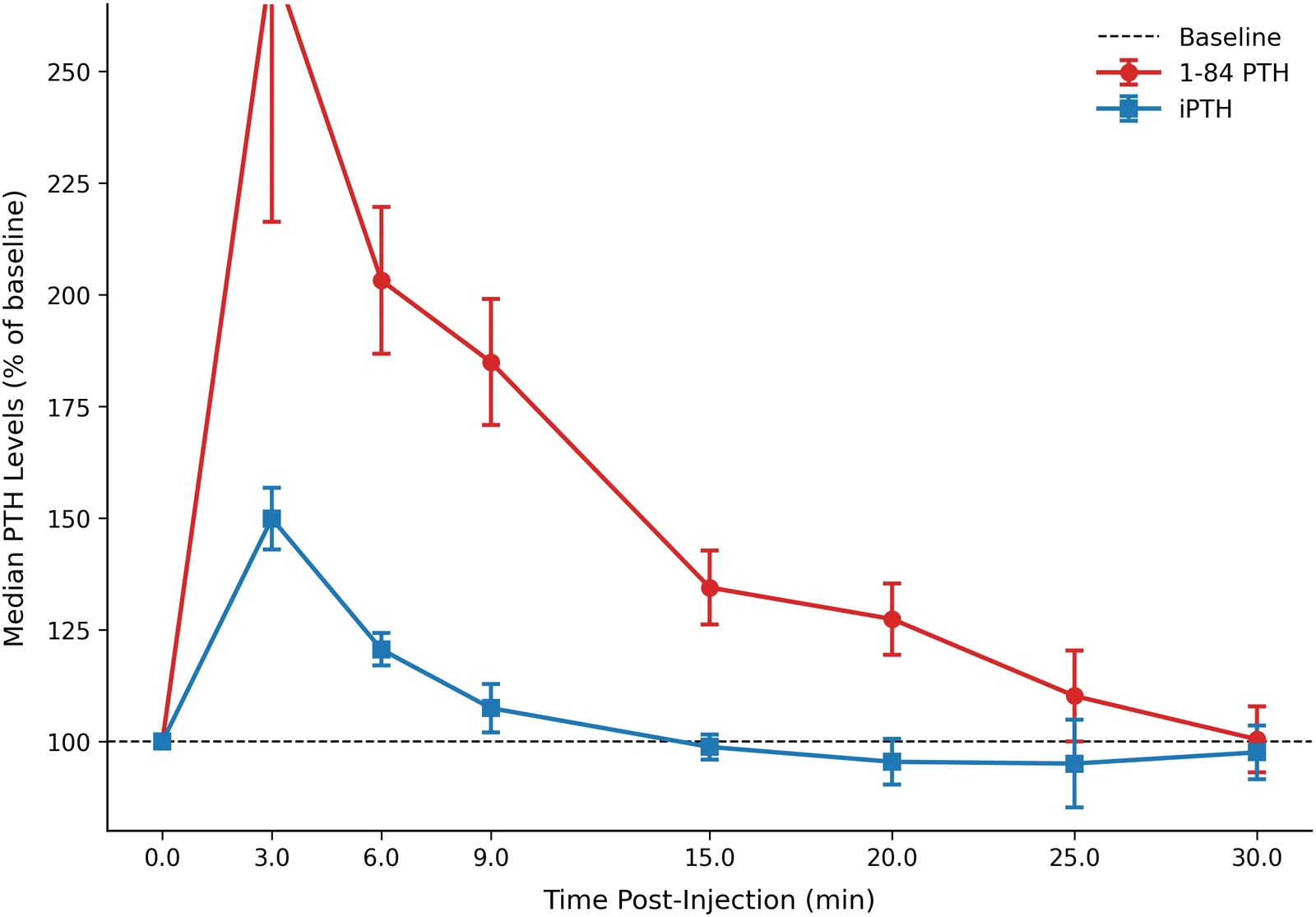
Relative kinetic profile of median 1–84 PTH and i-PTH levels post-injection. This image illustrates the median relative changes in concentration as a percentage of the baseline (Baseline = 100%, horizontal black dashed line) for two parathyroid hormone assays over a 30-minute observation period post-injection in the thyroidectomy cohort (n = 6): 1–84 PTH (red line with solid circles) and intact PTH (i-PTH, blue line with solid squares). Error bars indicate the dispersion of the data.

**Figure 3:**
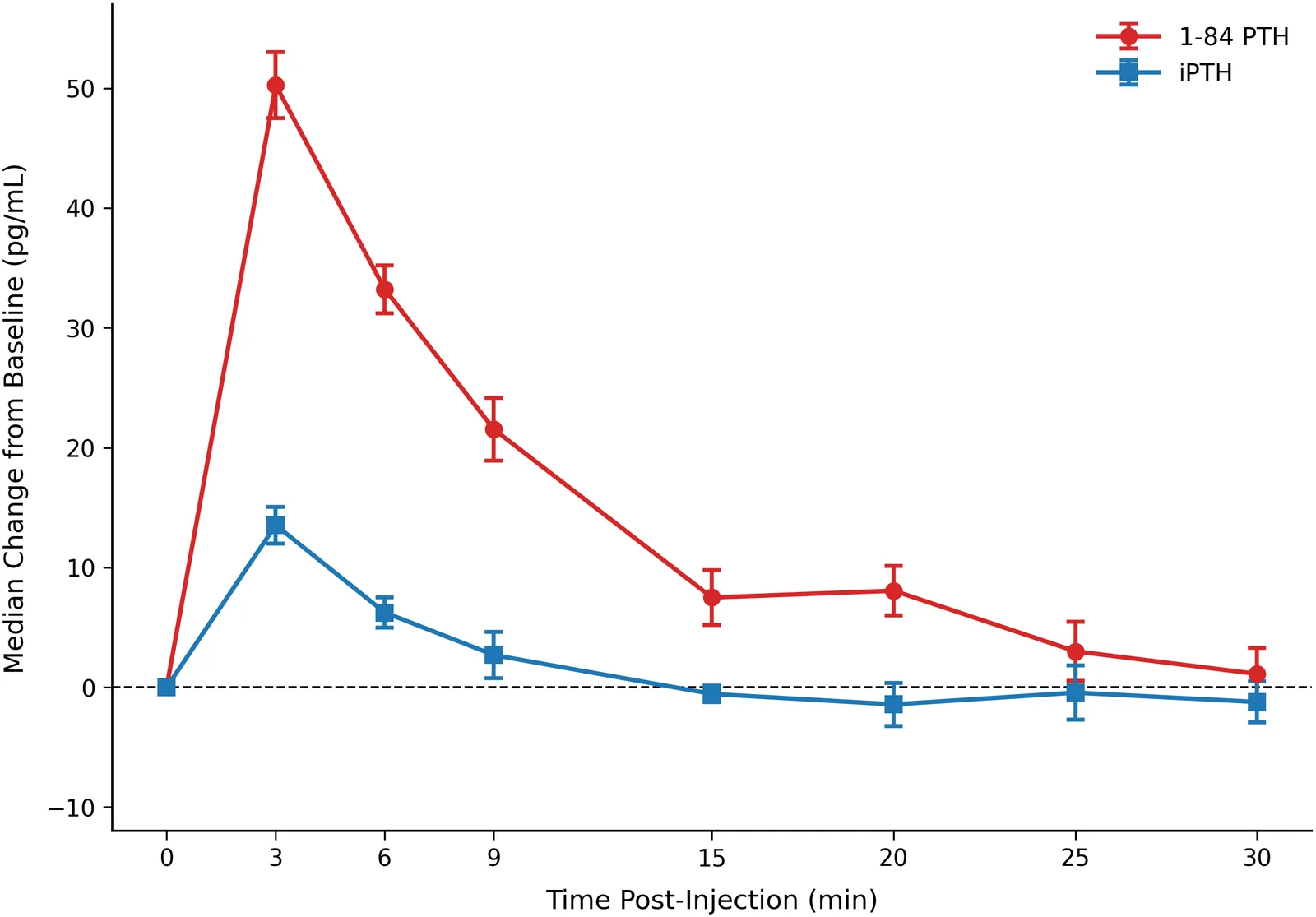
Dynamic profile of the median absolute change from baseline in 1–84 PTH and i-PTH assays post-injection. This image illustrates the median absolute change in concentration from baseline (Baseline = 0, horizontal black dashed line) for two parathyroid hormone assays over a 30-minute observation period post-injection in the thyroidectomy cohort (n = 6): 1–84 PTH (red line with solid circles) and intact PTH (i-PTH, blue line with solid squares). Error bars indicate the dispersion of the data.

Subsequently, measured concentrations declined in alignment with the hepatic clearance of the dye. By 15 minutes, second-generation i-PTH levels had largely stabilized near baseline (median change: -0.56 pg/mL; 98.3% of baseline), whereas third-generation PTH (1–84) maintained a residual median elevation of +7.49 pg/mL (122.9% of baseline). Between 20 and 30 minutes post-injection, both assays returned to baseline: i-PTH reached a median change of -0.21 pg/mL at 20 minutes and -1.23 pg/mL (96.6% of baseline) at 30 minutes (p > 0.05), while PTH (1–84) stabilized at +1.11 pg/mL (103.1% of baseline) at 30 minutes (p > 0.05). In this small cohort, these kinetic data show that the measured analytical bias diminished and values approached baseline within approximately 20 to 30 minutes; this interval should be interpreted as preliminary rather than as a validated biological washout period.

To evaluate whether this interference is assay-platform specific, parallel testing was conducted on paired pre- and post-ICG serum aliquots from a patient undergoing routine surgery across two automated systems utilizing distinct detection chemistries. On the electrochemiluminescence (ECLIA) platform (Roche Cobas e601), baseline i-PTH was 28.06 pg/mL, which surged to 370.5 pg/mL following intraoperative ICG administration. On the chemiluminescent microparticle immunoassay (CMIA) platform (Abbott Alinity i), baseline i-PTH was 29.6 pg/mL, which demonstrated a concordant increase to 382.0 pg/mL in the post-ICG sample. In this single-patient exploratory comparison, both systems showed marked post-ICG increases. This observation suggests that the phenomenon may not be restricted to a single analytical platform; however, the finding is preliminary and cannot establish platform independence.

Additional exploratory *in vitro* testing of three independent specimens showed non-uniform responses after ICG exposure: PTH measurements increased on Roche, were non-reportable because of read failure on Abbott, and decreased on Siemens Atellica IM (**Table 2**).

**Table 2:** Exploratory *in vitro* cross-platform PTH responses following ICG exposure.

Specimen	Platform	Baseline PTH (pg/mL)	Post-ICG PTH (pg/mL)	Analytical response
1	Roche Cobas e601	20.2	112	Increase
2	Roche Cobas e601	71.3	139	Increase
3	Roche Cobas e601	44.7	96.3	Increase
1	Abbott Alinity i	17.3	Not reportable*	Read failure
2	Abbott Alinity i	57.8	Not reportable*	Read failure
3	Abbott Alinity i	42.6	Not reportable*	Read failure
1	Siemens Atellica IM	16.1	13.4	Decrease
2	Siemens Atellica IM	71.3	48.7	Decrease
3	Siemens Atellica IM	43.5	24.0	Decrease

*For the additional *in vitro* experiment, 0.2 mL of ICG solution (2.5 mg/mL) was added to 4 mL of whole blood, mixed for 5 minutes, and centrifuged before analysis. For all three Abbott Alinity i specimens, error 1402 (“Unable to process test. Activated read failure”) occurred after ICG exposure and persisted after 10-fold dilution; therefore, no post-ICG PTH concentration was reportable. PTH, parathyroid hormone; ICG, indocyanine green.

Our findings show an immediate and transient post-ICG increase in measured PTH concentrations, with the largest change observed at 3 minutes and values approaching baseline over the subsequent 20 to 30 minutes in this small cohort. Because ICG fluorescence emission (~800–830 nm) does not overlap with the 620 nm ruthenium emission on ECLIA analyzers (11), simple spectral cross-talk appears less likely. One possible explanation is a nonspecific matrix or signal effect associated with macromolecular ICG–protein complexes formed at high intravascular concentrations (10, 12–15). Following injection, amphiphilic ICG binds extensively (>98%) to serum albumin and lipoproteins (10, 15), and such complexes could potentially perturb antibody binding or signal generation (13, 14). However, the present study did not directly investigate the molecular mechanism, and this explanation should therefore be regarded as a hypothesis rather than a demonstrated mechanism. The paired *in vitro* spiking experiments support a direct analytical contribution independent of surgical trauma or parathyroid manipulation. The third-generation whole PTH (1–84) assay showed a larger positive bias than the second-generation intact PTH (i-PTH) assay in the tested specimens, with inversion of the PTH (1–84)/i-PTH ratio. In addition, an exploratory single-patient comparison showed marked post-ICG increases on both Roche ECLIA and Abbott CMIA systems. This observation suggests that the phenomenon may not be restricted to one assay system, but broader platform independence cannot be inferred from a single patient. Several procedural observations reduce the likelihood that the early PTH increase was caused solely by local sampling-site contamination. All six participants had normal liver function test results and no history or diagnosis of liver cirrhosis. In addition, ICG was administered through a peripheral venous catheter, whereas serial PTH samples were obtained from an arterial catheter in the contralateral arm. Thus, direct contamination of the sampling line or transient retention of concentrated ICG within the venous system of the injection arm is unlikely to explain the early post-injection peak. Together with the reproducible positive bias observed in the independent *in vitro* spiking experiments, these findings support an ICG-associated analytical effect. Because the present study evaluated PTH only, no conclusions can be drawn regarding interference with other analytes. The additional exploratory cross-platform experiment showed non-uniform responses among the three evaluated systems and therefore does not support a platform-independent effect. This study has several limitations. The *in vivo* cohort was small (n = 6) and lacked a contemporaneous no-ICG control group. The cross-platform analysis involved limited specimens and were exploratory. The proposed interference mechanism was not directly tested and remains hypothetical. Finally, the observed return toward baseline within approximately 20 to 30 minutes should not be interpreted as a validated universal washout threshold. In summary, ICG can transiently distort ioPTH measurements and potentially mislead intraoperative interpretation. Whenever feasible, baseline ioPTH should be obtained before ICG administration. The observed 20-to-30-minute return toward baseline is preliminary and requires validation before a post-ICG sampling interval can be recommended.

## Clinical implications

The key practical implication is the timing of ioPTH sampling relative to ICG administration. Whenever feasible, baseline ioPTH should be obtained before ICG injection; post-ICG results should be interpreted cautiously because transient analytical distortion may occur.

## Data Availability

The raw data supporting the conclusions of this article will be made available by the authors, without undue reservation.
